# Sinusoidal Obstruction Syndrome (SOS) in Multiple Myeloma with Renal Failure

**DOI:** 10.1155/2018/5382852

**Published:** 2018-12-03

**Authors:** Urvi A. Shah, Sengottuvel Viswanathan, Beamon Agarwal, Aditi Shastri, Ioannis Mantzaris, Murali Janakiram, Noah Kornblum, Ira Braunschweig, Amit Verma, Yang Shi, John Reinus, Olga Derman

**Affiliations:** ^1^Department of Oncology, Montefiore Medical Center, 111 East 210th Street, Bronx, NY 10467, USA; ^2^Department of Pathology, Montefiore Medical Center, 111 East 210th Street, Bronx, NY 10467, USA; ^3^Department of Medicine, Division of Hepatology, Montefiore Medical Center, 111 East 210th Street, Bronx, NY 10467, USA

## Abstract

SOS is a rare complication of stem cell transplantation and has significant morbidity and mortality. We present three cases of SOS and highlight underlying risk factors for its development, such as impaired clearance of alkylating agents (especially melphalan) in patients with renal failure and prolonged infection. Although, melphalan and cyclophosphamide cause SOS less commonly than alkylating agents such as busulfan, physicians must use caution when administering these drugs to patients with underlying comorbidities such as renal failure that may increase the likelihood of development of SOS. This is due to unpredictable pharmacokinetics in patients with renal failure and therefore close drug monitoring is required. With the recent FDA approval of defibrotide in 2016, outcomes of SOS have improved and physician awareness is important for prompt diagnosis and treatment.

## 1. Introduction

Sinusoidal obstruction syndrome (SOS), previously known as venoocclusive disease (VOD), is a rare life-threatening condition seen most commonly after hematopoietic stem cell transplantation (HSCT). Patients with SOS were managed with supportive care until 2016, when defibrotide was FDA approved for treatment of this disorder. We present three cases of SOS and highlight underlying risk factors for its development, such as impaired clearance of alkylating agents in patients with renal failure and prolonged infection.

## 2. Case Presentation

### 2.1. Case 1

**Figure 1:**
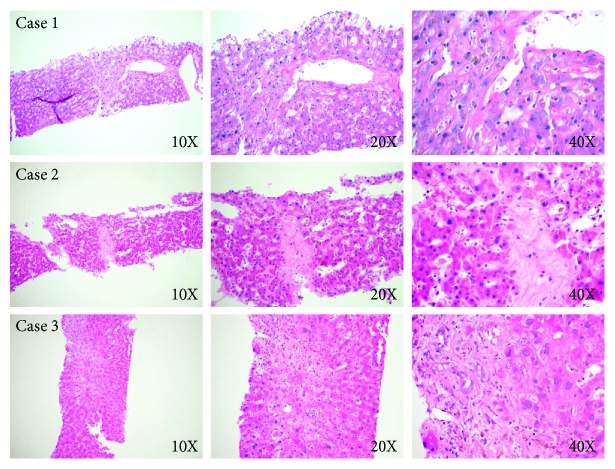
Diagnosis of SOS on liver biopsy in case 1, 2, and 3. Case 1. Hematoxylin and eosin-stained section of the liver biopsy shows some portal areas with sparse inflammation comprising predominantly of lymphocytes and rare plasma cells, with fibrous expansion of most portal areas. Bile ducts show mild dystrophy and regenerative changes in some portal tracts. The hepatocyte shows dropout of hepatocytes and acidophilic bodies predominantly involving centrilobular areas with intracanalicular and intrahepatic cholestasis. Case 2. Hematoxylin and eosin-stained section of the liver biopsy shows preserved lobular architecture with patchy fibrosis within sinusoids and marked central venular fibrosis. Portal tracts show mild portal fibrosis and focal ductular reaction. Some of the bile ducts show cytoplasmic vacuolation, disordered nuclear polarity, and occasional mononuclear inflammatory infiltrates within the bile duct epithelium. Case 3. Hematoxylin and eosin-stained section of the liver biopsy shows foci of centrilobular hepatocyte drop-out, cholestasis, and portal areas with ductular reaction. A few central veins show partial to near complete occlusion and pericellular fibrosis.

A 52-year-old woman with kappa light chain multiple myeloma who was noncompliant with treatment presented one month after diagnosis with plasma cell leukemia and end-stage renal disease (ESRD) requiring hemodialysis (HD). She was treated with one cycle of bortezomib, doxorubicin, and dexamethasone and then, due to noncompliance, switched to four cycles of bortezomib, cyclophosphamide, and dexamethasone (CyBorD). She was mobilized with filgrastim followed by an autologous HSCT and melphalan (140 mg/m^2^) therapy. After transplantation, she developed Enterobacter and MRSA bacteremia that was treated with colistin and vancomycin for 14 days. Her total serum bilirubin level began to rise on treatment day seven and peaked at 7.8 mg/dl on treatment day 15. She also developed hepatomegaly, ascites, and had a ≥ 10% weight gain. Liver biopsy on treatment day 14 showed SOS (Figure 1). She was treated with ursodiol and subsequently recovered.

### 2.2. Case 2

A 53-year-old man with kappa light chain multiple myeloma and ESRD on HD was treated with five cycles of CyBorD followed by high-dose cyclophosphamide mobilization and autologous HSCT with melphalan 140 mg/m^2^ therapy. His subsequent course was complicated by neutropenic fever with Streptococcus sanguinis bacteremia treated with vancomycin, cefazolin, and metronidazole. He had no history of liver disease, but his total serum bilirubin level was 3.4 mg/dl at the time of transplantation and peaked on treatment day 24 at 22 mg/dl. Imaging revealed ascites. Liver biopsy on treatment day 16 was consistent with SOS (Figure 1). He was treated with ursodiol and died on treatment day 25 before he could receive defibrotide under compassionate use.

### 2.3. Case 3

A 57-year-old man with kappa light chain multiple myeloma was treated for approximately two years with CyBorD. The patient had ESRD and required HD. CyBorD treatment was stopped when he was diagnosed with *Staphylococcus epidermidis* endocarditis that was treated with vancomycin followed by ampicillin-sulbactam for one month. His myeloma subsequently relapsed and stem cells were collected with filgrastim and plerixafor mobilization. Five days after collection, he presented with jaundice and a total bilirubin level of 12 mg/dl which peaked at 15.9 mg/dl one month later. Liver histology showed SOS without myeloma (Figure 1). The patient was treated with ursodiol. He was then lost to follow-up and HSCT was not performed.

These three patients with multiple myeloma and ESRD were treated with alkylating agents and autologous HSCT; each had prolonged infection and developed severe SOS in case 1, very severe SOS in case 2, and moderate SOS in case 3 based on proposed grading of SOS severity by EBMT criteria [1] (Table 1). Although case 3 did not have a HSCT, he was exposed to prolonged periods of alkylating agents and antibiotics such as vancomycin in the setting of a serious infection and renal dysfunction which are all underlying risk factors (Table 2). Patients with renal failure are at a greater than normal risk of developing drug-induced SOS because of altered pharmacokinetics, with marked interindividual variation [2].

## 3. Discussion

SOS is a rare disease with significant morbidity and mortality. In the pretransplantation era, it was rarely seen and usually occurred in association with bush-tea consumption, high-dose chemotherapy, or treatment with other drugs. [3] The first fatal posttransplantation case of SOS was reported in 1979 in a patient with refractory acute leukemia [4]. In 135 reports of patients with autologous or allogeneic HSCTs, the mean incidence of SOS was 13.7% [11.5% (1979–1994) vs 14.6% (1994–2007)] [5]. The rise in incidence was attributed to transplantation of older patients, who received multiple therapies including multiple alkylating agents [5].

SOS conventionally is diagnosed based on clinical criteria (modified Seattle [6] and Baltimore [7] criteria) and occurs within three weeks of transplantation in the majority of cases. Moderate to severe SOS is associated with painful hepatomegaly, rapid weight gain, ascites, and jaundice. If SOS progresses to multiorgan failure, mortality is as high as 84% [5]. Mortality from SOS has declined since the introduction of defibrotide in 1997, the only significant change in treatment for these patients in the last 20 years [8]. Recently in March 2016, the U.S. FDA approved defibrotide sodium, a polydeoxyribonucleic acid, to treat hepatic VOD in patients with kidney or lung abnormalities after a HSCT and a delay in administration is associated with worse outcomes. The approval was based on survival at treatment day 100 after HSCT in two prospective clinical trials (phase II [9] and III [10]) as well as an expanded access study [11]. The treatment day 100 survival rates in these studies were 44% [9], 38% [10], and 45% [11], respectively, as compared to older published reports and analyses of patient level data prior to defibrotide availability where it is significantly lower at 21–31% [12].

Multiple risk factors predispose patients to develop SOS [13]. Most commonly implicated are the conditioning regimen and the type of HSCT. Underlying liver dysfunctions, such as abnormal liver enzyme levels and hepatitis B or C infections, are independent risk factors for the development of SOS after HSCT [14]. Treatment with antimicrobials, such as vancomycin, amphotericin, and acyclovir, also is associated with SOS [14, 15]; their use is considered an indirect marker for persistent fever and infection [14].

**Figure 2:**
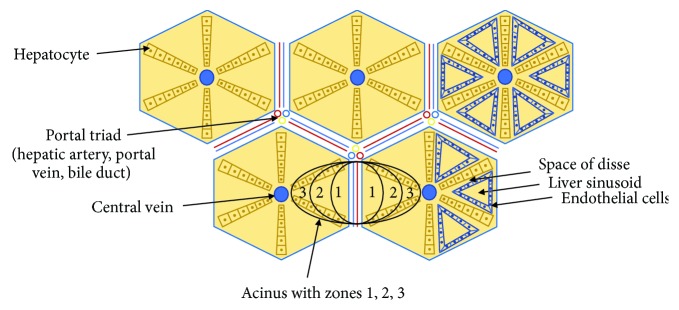
Schematic representation of the hepatic lobule and acinus. The acinus is the physiological unit of the liver and is divided into three zones, according to distance from the afferent arterial supply. Sinusoids are distensible vascular channels bounded circumferentially by hepatocytes and lined with SEC. As blood flows through the sinusoids, plasma is filtered through pores in the endothelium into the space between endothelium and hepatocytes (the “space of Disse”) as lymph. In SOS, obstruction to the sinusoids develops in zone 3. This leads to pathological increased pressure in the sinusoids and an increase in the rate of lymph production, which accumulates in the abdominal cavity as ascites.

There is a lower mean incidence of SOS after autologous HSCT than after allogeneic HSCT (8.7% vs 12.9%) [5]. Conditioning regimens determine the overall risk of developing SOS; several alkylating agents given in high doses have been associated with development of SOS in this setting [3, 16]. Alkylating-agent metabolites have been shown *in vitro* to deplete hepatic glutathione levels, inducing oxidative stress [17]. SOS is believed to be caused by cytoreductive injury to hepatocytes and endothelial cells in zone three of the liver acinus. This is strongly influenced by factors that induce the release of tumor necrosis factor-*α* (TNF-*α*) leading to coagulation with obstruction of hepatic sinusoids and venules [15] (Figure 2).

The most commonly implicated alkylating agent for the development of SOS is busulfan. Unpredictable absorption and hepatic first-pass metabolism of oral busulfan led to the development of IV busulfan, which is associated with a significantly lower incidence of SOS [18]. Use of Bayesian individualization of busulfan dosage also may lower the SOS rate [19].

Cyclophosphamide, another alkylator, may cause elevation of serum bilirubin levels, SOS, and mortality in direct proportion to drug exposure [20]. The International Myeloma Working Group does not recommend cyclophosphamide dose reduction in myeloma patients with renal impairment, given that it is relatively safe [21]. Although cyclophosphamide dose adjustment is not necessary in patients with moderate renal impairment, this drug must be used with caution in persons with severe renal impairment because studies have shown that renal impairment causes decreased excretion of cyclophosphamide and its metabolites, increasing the risk of toxicity [22–25].

Standard-dose melphalan is rarely associated with hepatotoxicity [26]. Melphalan is metabolized by hydrolysis and dechlorination by hepatic cytochrome P450. At high doses, melphalan causes transient elevations of liver enzyme levels [27]. Preclinical studies show that melphalan induces caspase-dependent apoptosis of hepatocytes by increasing membrane-bound TNF in Kupffer cells [28]. Direct cytotoxic injury causing sinusoidal endothelial cell death and extrusion into sinusoids, with subsequent sinusoidal and hepatic venular obstruction is another potential mechanism (https://livertox.nih.gov/Melphalan.htm) (Figure 2).

Although melphalan is eliminated from plasma primarily by chemical hydrolysis to noncytotoxic monohydroxy and dihydroxy metabolites [29], it is both secreted and reabsorbed by the renal tubules. Therefore, its clearance is renal-function dependent [2]. For high-dose melphalan (200 mg/m^2^), a reduced dose of 140 mg/m^2^ is used when the creatinine clearance is less than 60 ml/min [30]. Pharmacokinetic studies have demonstrated large interindividual variations (10-fold) in melphalan excretion [27, 31–33]. Some early studies recommended dosing based on the pharmacokinetic response to a test dose to minimize toxicity [34]. Given the interindividual variability in elimination, optimal melphalan dosing in patients with a creatinine clearance less than 60 ml/min is difficult [2], possibly exposing these patients to a greater risk of hepatotoxicity and hematologic toxicity [35]. Melphalan is not cleared to any significant degree by hemodialysis; therefore dose adjustment is dependent on renal function and not dialysis status.

High-dose melphalan treatment in preparation for HSCT, however, has rarely been associated with SOS. A study that assessed the safety of autologous HSCT in six patients with multiple myeloma and chronic renal failure noted that one patient who was treated with busulfan and melphalan at 80 mg/m^2^ as a preconditioning regimen developed SOS on treatment day 15 [33]. Cases of severe SOS in patients with normal renal function have been reported after preconditioning treatment with melphalan 200 mg/m^2^ in one patient who received an autologous HSCT [36] and in two patients after tandem HSCTs [37, 38].

In 2007 the Spanish Myeloma Group studied patients with newly diagnosed multiple myeloma treated with a total of six cycles of alternating VBMCP/VBAD chemotherapy followed by oral busulfan and melphalan 140 mg/m^2^ and autologous HSCT. During two years of follow-up, a number of clinical episodes resembling SOS were seen. Consequently, the protocol was modified, and patients were treated with melphalan 200 mg/m^2^ only. Three years later, after a total of 734 patients had undergone a first autologous HSCT, the authors noted an 8% incidence of SOS (2% mortality) in patients treated with busulfan and melphalan as compared to a 0.4% incidence (0.2% mortality) in the patients treated with melphalan alone. This trial showed that melphalan alone can cause SOS but this complication is much more common when melphalan is used in combination with busulfan [39].

## 4. Conclusion

Although melphalan and cyclophosphamide cause SOS less commonly than alkylating agents such as busulfan, physicians must use caution when administering these drugs to patients with underlying renal failure, liver disease, or prolonged infection and when they are given along with alkylating drugs. Close monitoring of patients with underlying risk factors for the development of SOS and prompt treatment with defibrotide if SOS is suspected can decrease the morbidity and mortality associated with this diagnosis.

## Figures and Tables

**Table 1:** Three cases of IgG kappa light chain multiple myeloma.

Case	Chemo	Auto SCT	Risk factors	EBMT grading	Treatment	Outcome
1: 52 F	CyBorD	Melphalan	ESRD, cyclophosphamide, melphalan, infection, vancomycin	Severe	Ursodiol	Recovery
2: 53 M	CyBorD	Melphalan	ESRD, cyclophosphamide, liver disease, melphalan, infection, vancomycin	Very severe	Ursodiol	Death
3: 57 M	CyBorD	—	ESRD, cyclophosphamide, infection, vancomycin	Moderate	Ursodiol	Lost to follow-up

**Table 2:** Well-known risk factors for SOS (adapted from [1, 3]).

*Transplant-related factors*
Unrelated donor
HLA-mismatched donor
Non-T-cell-depleted transplant
Myeloablative-conditioning regimen
Oral or high-dose busulfan-based regimen
High-dose total body irradiation-based regimen
Second HSCT
GVHD prophylaxis regimen
*Patient and disease-related factors*
Older age
Karnofsky score below 90%
Metabolic syndrome
Female receiving norethisterone
Advanced disease (beyond second CR or relapse/refractory)
Thalassemia
Genetic factors (GSTM1 polymorphism, C282Y allele, MTHFR 677CC/1298CC haplotype)
Infection/antibiotic/antiviral use (vancomycin, acyclovir)
Renal dysfunction due to impaired drug clearance
*Hepatic related*
Elevated liver function tests
Cirrhosis
Active viral hepatitis
Abdominal or hepatic irradiation
Previous use of gemtuzumab ozogamicin or inotuzumab ozogamicin
Hepatotoxic drugs
Iron overload

## References

[1] Mohty M., Malard F., Abecassis M. (2016). Revised diagnosis and severity criteria for sinusoidal obstruction syndrome/veno-occlusive disease in adult patients: a new classification from the European Society for Blood and Marrow Transplantation. *Bone Marrow Transplantation*.

[2] Esma F., Salvini M., Troia R., Boccadoro M., Larocca A., Pautasso C. (2017). Melphalan hydrochloride for the treatment of multiple myeloma. *Expert Opinion on Pharmacotherapy*.

[3] Dalle J. H., Giralt S. A. (2016). Hepatic veno-occlusive disease after hematopoietic stem cell transplantation: risk factors and stratification, prophylaxis, and treatment. *Biology of blood and marrow transplantation : journal of the American Society for Blood and Marrow Transplantation*.

[4] Jacobs P., Miller J. L., Uys C. J., Dietrich B. E. (1979). Fatal veno-occlusive disease of the liver after chemotherapy, whole-body irradiation and bone marrow transplantation for refractory acute leukaemia. *South African medical journal*.

[5] Coppell J. A., Richardson P. G., Soiffer R. (2010). Hepatic veno-occlusive disease following stem cell transplantation: incidence, clinical course, and outcome. *Biology of blood and marrow transplantation*.

[6] McDonald G. B., Sharma P., Matthews D. E., Shulman H. M., Donnall Thomas E. (1984). Venocclusive disease of the liver after bone marrow transplantation: diagnosis, incidence, and predisposing factors. *Hepatology*.

[7] Jones R. J., Lee K. S. K., Beschorner W. E. (1987). Venoocclusive disease of the liver following bone marrow transplantation. *Transplantation*.

[8] Carreras E., Díaz-Beyá M., Rosiñol L., Martínez C., Fernández-Avilés F., Rovira M. (2011). The incidence of veno-occlusive disease following allogeneic hematopoietic stem cell transplantation has diminished and the outcome improved over the last decade. *Biology of blood and marrow transplantation*.

[9] Richardson P. G., Soiffer R. J., Antin J. H. (2010). Defibrotide for the treatment of severe hepatic veno-occlusive disease and multiorgan failure after stem cell transplantation: a multicenter, randomized, dose-finding trial. *Biology of Blood and Marrow Transplantation*.

[10] Richardson P. G., Riches M. L., Kernan N. A. (2016). Phase 3 trial of defibrotide for the treatment of severe veno-occlusive disease and multi-organ failure. *Blood*.

[11] Richardson P. G., Smith A. R., Triplett B. M. (2017). Defibrotide for patients with hepatic veno-occlusive disease/sinusoidal obstruction syndrome: interim results from a treatment IND study. *Biology of Blood and Marrow Transplantation*.

[12] Richardson P. G., Smith A. R., Triplett B. M. (2017). Earlier defibrotide initiation post-diagnosis of veno-occlusive disease/sinusoidal obstruction syndrome improves day +100 survival following haematopoietic stem cell transplantation. *British Journal of Haematology*.

[13] Mohty M., Malard F., Abecassis M. (2015). Sinusoidal obstruction syndrome/veno-occlusive disease: current situation and perspectives-a position statement from the European Society for Blood and Marrow Transplantation (EBMT). *Bone Marrow Transplantation*.

[14] McDonald G. B., Hinds M. S., Fisher L. D. (1993). Veno-occlusive disease of the liver and multiorgan failure after bone marrow transplantation: a cohort study of 355 patients. *Annals of Internal Medicine*.

[15] Shulman H. M., Hinterberger W. (1992). Hepatic veno-occlusive disease--liver toxicity syndrome after bone marrow transplantation. *Bone Marrow Transplantation*.

[16] Tsirigotis P. D., Resnick I. B., Avni B. (2014). Incidence and risk factors for moderate-to-severe veno-occlusive disease of the liver after allogeneic stem cell transplantation using a reduced intensity conditioning regimen. *Bone Marrow Transplantation*.

[17] DeLeve L. D., Wang X. (2000). Role of oxidative stress and glutathione in busulfan toxicity in cultured murine hepatocytes. *Pharmacology*.

[18] Kashyap A., Wingard J., Cagnoni P. (2002). Intravenous versus oral busulfan as part of a busulfan/cyclophosphamide preparative regimen for allogeneic hematopoietic stem cell transplantation: decreased incidence of hepatic venoocclusive disease (HVOD), HVOD-related mortality, and overall 100-day mortality. *Biology of blood and marrow transplantation*.

[19] Brice K., Valerie B., Claire G. (2008). Risk-adjusted monitoring of veno-occlusive disease following Bayesian individualization of busulfan dosage for bone marrow transplantation in paediatrics. *Pharmacoepidemiology and Drug Safety*.

[20] McDonald G. B., Slattery J. T., Bouvier M. E. (2003). Cyclophosphamide metabolism, liver toxicity, and mortality following hematopoietic stem cell transplantation. *Blood*.

[21] Dimopoulos M. A., Sonneveld P., Leung N. (2016). International myeloma working group recommendations for the diagnosis and management of myeloma-related renal impairment. *Journal of Clinical Oncology*.

[22] Ekhart C., Kerst J. M., Rodenhuis S., Beijnen J. H., Huitema A. D. R. (2009). Altered cyclophosphamide and thiotepa pharmacokinetics in a patient with moderate renal insufficiency. *Cancer Chemotherapy and Pharmacology*.

[23] Juma F. D., Rogers H. J., Trounce J. R. (1981). Effect of renal insufficiency on the pharmacokinetics of cyclophosphamide and some of its metabolites. *European Journal of Clinical Pharmacology*.

[24] Mouridsen H. T., Jacobsen E. (1975). Pharmacokinetics of cyclophosphamide in renal failure. *Acta Pharmacologica et Toxicologica*.

[25] Bramwell V., Calvert R. T., Edwards G., Scarffe H., Crowther D. (1979). The disposition of cyclophosphamide in a group of myeloma patients. *Cancer Chemotherapy and Pharmacology*.

[26] King P. D., Perry M. C. (2001). Hepatotoxicity of chemotherapy. *The Oncologist*.

[27] Lee J. L., Gooley T., Bensinger W., Schiffman K., McDonald G. B. (1999). Veno-occlusive disease of the liver after busulfan, melphalan, and thiotepa conditioning therapy: incidence, risk factors, and outcome. *Biology of Blood and Marrow Transplantation*.

[28] Kresse M., Latta M., Kunstle G. (2005). Kupffer cell-expressed membrane-bound TNF mediates melphalan hepatotoxicity via activation of both TNF receptors. *Journal of Immunology*.

[29] Alberts D. S., Chang S. Y., Chen H. S. G. (1979). Kinetics of intravenous melphalan. *Clinical Pharmacology and Therapeutics*.

[30] Dimopoulos M. A., Terpos E., Chanan-Khan A. (2010). Renal impairment in patients with multiple myeloma: a consensus statement on behalf of the International Myeloma Working Group. *Journal of clinical oncology*.

[31] Tricot G., Alberts D. S., Johnson C. (1996). Safety of autotransplants with high-dose melphalan in renal failure: a pharmacokinetic and toxicity study. *Clinical cancer research*.

[32] Kintzel P. E., Dorr R. T. (1995). Anticancer drug renal toxicity and elimination: dosing guidelines for altered renal function. *Cancer Treatment Reviews*.

[33] Tosi P., Zamagni E., Ronconi S. (2000). Safety of autologous hematopoietic stem cell transplantation in patients with multiple myeloma and chronic renal failure. *Leukemia*.

[34] Tranchand B., Ploin Y. D., Minuit M. P. (1989). High-dose melphalan dosage adjustment: possibility of using a test-dose. *Cancer Chemotherapy and Pharmacology*.

[35] Carlson K., Hjorth M., Knudsen L. M., The Nordic Myeloma Study Group (2005). Toxicity in standard melphalan-prednisone therapy among myeloma patients with renal failure--a retrospective analysis and recommendations for dose adjustment. *British Journal of Haematology*.

[36] Dolai T. K., Nataraj K. S., Bhattacharya M., Ghosh M. K. (2012). Veno-occlusive disease following high dose melphalan. *Indian journal of hematology & blood transfusion*.

[37] Labidi S. I., Sebban C., Ghesquières H., Nicolas E. V., Biron P. (2008). Hepatic veno-occlusive disease after tandem autologous stem cell transplantation conditioned by melphalan. *International Journal of Hematology*.

[38] Harousseau J. L., Milpied N., Laporte J. P. (1992). Double-intensive therapy in high-risk multiple myeloma. *Blood*.

[39] Carreras E., Rosiñol L., Terol M. J. (2007). Veno-occlusive disease of the liver after high-dose cytoreductive therapy with busulfan and melphalan for autologous blood stem cell transplantation in multiple myeloma patients. *Biology of blood and marrow transplantation*.

